# A 30-InDel Assay for Genetic Variation and Population Structure Analysis of Chinese Tujia Group

**DOI:** 10.1038/srep36842

**Published:** 2016-11-11

**Authors:** Chunmei Shen, Bofeng Zhu, Tianhua Yao, Zhidan Li, Yudang Zhang, Jiangwei Yan, Bo Wang, Xiaohua Bie, Fadao Tai

**Affiliations:** 1 Blood Center of Shaanxi Province, Xi’an, Shaanxi 710061, P. R. China; 2 Institute of Brain and Behavioral Sciences, College of Life Sciences, Shaanxi Normal University, Xi’an, Shaanxi 710062, P. R. China; 3 Department of Forensic Genetics, School of Forensic Medicine, Southern Medical University, Guangzhou 510515, P. R. China; 4 Key Laboratory of Shaanxi Province for Craniofacial Precision Medicine Research, College of Stomatology, Xi’an Jiaotong University, Xi’an, Shaanxi 710004, P. R. China; 5 Clinical Research Center of Shaanxi Province for Dental and Maxillofacial Diseases, College of Stomatology, Xi’an Jiaotong University, Xi’an, Shaanxi 710004, P. R. China; 6 Department of General Dentistry, Stomatological Hospital, Xi’an Jiaotong University, Xi’an, Shaanxi 710004, P. R. China; 7 Department of Endodontics, Stomatological Hospital, Xi’an Jiaotong University, Xi’an, Shaanxi 710004, P. R. China; 8 Institute of Forensic Science of Anhui Public Security Department, Hefei, Anhui 230061, P. R. China; 9 Key Laboratory of Genome Sciences, Beijing Institute of Genomics, Chinese Academy of Sciences, Beijing 100101, P. R. China; 10 College of Biological Technology, Xi’an University, Xi’an, Shaanxi 710065, P. R. China; 11 Department of Neurosurgery, Hong-hui Hospital, Xi’an Jiaotong University College of Medicine, Xi’an, Shaanxi 710054, P. R. China

## Abstract

In the present study, thirty autosomal insertion and deletion polymorphic loci were simultaneously amplified and genotyped in a multiplex system, and their allelic frequencies as well as several forensic parameters were obtained in a sample of 236 unrelated healthy Tujia individuals. All the loci were in Hardy-Weinberg equilibrium after applying a Bonferroni correction and all pair-wise loci showed no significant linkage disequilibrium. These loci were observed to be relatively informative and discriminating, quite efficient for forensic applications. Allelic frequencies of 30 loci were compared between the Tujia group and other reference populations, and the results of analysis of molecular variance indicated the Tujia group showed the least significant differences with the Shanghai Han at one locus, and the most with Central Spanish population at 22 loci. We analyzed the population genetic structure by the principal component analysis, the clustering of STRUCTURE program and a Neighbor-Joining tree, and then evaluated the genetic relationships among Tujia and other 15 populations.

Short tandem repeats (STRs) have become popular DNA markers in forensic DNA labs for more than 20 years and have been proved to possess several benefits, which make them especially suitable to identify victims, perpetrators, missing persons, and for kinship testing and population genetic analysis^1^,^2^,^3^,^4^,^5^. However, there were some potential limitations of STRs in forensic applications because of its relatively high mutation rate, long amplicon size, and the deficiency in the analysis of highly degraded DNA samples and complex kinship cases. In recent years, a novel genetic marker: insertion and deletion polymorphisms (InDels) dispersing through the human genome showed some advantages, such as short amplicon size, low mutation rate, and practicability of being genotyped in the present forensic DNA lab platforms^6^,^7^,^8^, which were useful for forensic DNA applications (Supplementary method for STR applications), population genetics^9^,^10^,^11^,^12^, and biogeographic ancestry analysis^13^,^14^,^15^. Population genetic and forensic validation studies have been performed using the Qiagen Investigator DIPplex^®^ reagent including 30 autosomal InDel loci plus amelogenin locus, and population data of Chinese Han, Tibetan, Uigur, Kazak, She, Xibe and Yi populations have been reported in previous studies^9^,^10^,^16^,^17^,^18^. In the present study, we firstly reported the population genetic data of 30 InDels in Chinese Tujia ethnic group, evaluated their usefulness in the field of forensic sciences, analyzed the interpopulation differentiations, and retraced the genetic background of the Tujia group by the population structure construction, principal component analysis, phylogenetic tree and some other analyses.

## Materials and Methods

### Ethical statement and population samples

Bloodstain samples were randomly collected from 236 unrelated healthy Tujia individuals in Enshi Tujia and Miao Autonomous Prefecture of Hubei province, China. The study was conducted in accordance with the human and ethical research principles of Xi’an Jiaotong University Health Science Center and approved by the ethics committee of Xi’an Jiaotong University Health Science Center. We have obtained written informed consent from all volunteers for the purpose of research. The investigation was conducted in order to ensure that any two individuals didn’t share a common ancestry within at least three previous generations; all individuals were born and lived in the same prefecture; and their ancestors married no any other ethnic people.

### DNA extraction, co-amplification and genotyping

Genomic DNA was extracted from bloodstain cards by using the Chelex^®^ method (Solarbio, Beijing, China) according to the manufacturer’s instructions^19^. About 0.5–1.0 ng genomic DNA was used for amplification with a 25 ul reaction volume. PCR amplification for 30 InDel loci and Amelogenin locus was performed in a single multiplex reaction using the DIPplex Investigator reagent (Qiagen, Hilden, Germany), which was prepared on a GeneAmp^®^ PCR System 9700 thermal cycler (Applied Biosystems, Foster City, CA, USA) under the recommended reaction condition. PCR products of all loci were separated and detected by capillary electrophoresis on the ABI 3500 Genetic Analyzer (Applied Biosystems). Genotyping of InDel loci was analyzed using the BTO 550 (Qiagen) as internal lane standard and by GeneMapper^®^ ID software v3.2 (Applied Biosystems). Experiments were carried out according to the kit control and the ISO 17025 standard in this study.

### Statistical analyses

Hardy-Weinberg equilibrium (HWE), allelic frequencies and forensic statistical parameters of 30 InDels were calculated by the modified powerstat (version1.2) spreadsheet (Promega, Madison, WI, USA). Linkage disequilibrium (LD) analysis for all pair-wise InDel loci was performed using the SNPAnalyzer v2.0 (Istech, South Korea)^20^. *Fst* and *p* values for pairwise interpopulation comparisons were calculated based on allele frequencies of 30 InDels by analysis of molecular variance (AMOVA) performed with ARLEQUIN version 3.1 software (http://cmpg.unibe.ch/software/arlequin3). Principal component analysis (PCA) in two forms and phylogenetic reconstruction were employed in MATLAB 2007a (MathWorks Inc., USA), R statistical software v3.0.2^21^ and genetic distance and phylogenetic analysis (DISPAN) program (http://pritch. bsd.uchicago.edu), respectively. The detailed population genetic structure was performed with the STRUCTURE program v2.2 (http://pritch.bsd.uchicago.edu.) to analyze the structure of Tujia and the other populations previously published based on the same 30 InDels.

## Results and Discussion

### Allele diversities within group

Probability values for Hardy-Weinberg equilibrium tests for 29 InDel loci ranged from 0.0669 (HLD40) to 0.9987 (HLD97), and *p* < 0.05 was only observed at the HLD88 locus (*p* = 0.0382). *P* values were adjusted after applying a Bonferroni correction for all 30 InDel loci analyzed and *P* > 0.00167 was considered statistically insignificant. Then, the genotype frequency data for all loci showed no deviations from HWE expectations in the sample of Tujia group. Allelic frequencies and forensic statistical parameters of 30 InDels based on the raw genotype (shown in Supplementary Table 1) were shown in Table 1. Allelic frequencies of deletion allele at the 30 InDel loci ranged from 0.0445 to 0.9089 in the group, with a mean value of 0.4939. The observed (HO) and expected heterozygosities (HE) ranged from 0.0890 (HLD118) to 0.5381(HLD92); and 0.0850 (HLD118) to 0.4985(HLD136), with a mean value of 0.4028 and 0.4073, respectively. Twenty-four InDel loci had power of discrimination (PD) values greater than 0.5, except the six loci: HLD39, HLD64, HLD81, HLD99, HLD111, and HLD118 loci. The values of the power of exclusion (PE), the matching probability (MP), the typical paternity index (TPI), and the polymorphic information content (PIC) ranged from 0.0067 to 0.2231, 0.3524 to 0.8379, 0.5488 to 1.0826 and 0.0814 to 0.3742, respectively. The lowest HO, HE, PIC, TPI, PD and PE were observed at HLD 118 locus, and this locus was also found with the lowest polymorphism in other previously studied groups^10^. The combined power of exclusion (CPE) and discrimination (CPD) at the 30 InDel loci in the Tujia group were 0.9860 and 0.9999999999761, respectively; combined matching probability (CMP) value of 30 InDels in the group was 2.3894 × 10^−11^, higher than that in our previous study which reached 1.10974 × 10^−19^ of 21 autosomal STRs in Tujia group^11^. According to our calculation, the value of CMP combining 30 InDels with 21 autosomal STRs reached 2.652 × 10^−30^. These data suggested that the panel of 30 InDel loci could be a valid supplement to the routine detection of autosomal STRs in forensic cases.

### Linkage disequilibrium tests

Linkage disequilibrium tests of these pairwise InDels were analyzed using the SNPAnalyzer version 2.0 and obtained several indexes: LOD, *r^2^* and |D’|. As shown in Supplementary Fig. 1, no strong linkage disequilibrium between two different InDels was observed in a total of 435 interclass correlation tests (data not shown) with the values of *r^2^* less than 0.8, and no crimson box was coated by a thick black curve. The present LD tests suggested that 30 InDels were independent for the following statistical analyses, and also suited for forensic cases in the Tujia group.

### Genetic divergences

Genetic distance is a measure method of the genetic divergence between different populations, used for understanding the origin of biodiversity and reconstructing the history of different ethnic groups^22^. We measured the Nei’s *D*_*A*_ distance by examining the differences between allelic frequencies at the same set of 30 InDel loci of different populations. *D*_*A*_ distances between the 16 groups with each other based on allelic frequencies of the 30 InDel loci were shown in Table 2. Short genetic distances were found between the Tujia group and Shanghai Han^17^, Guangdong Han^18^, South Korean^23^, Beijing Han^9^, Xibe^10^, She^17^, Tibetan^9^, and Yi^16^ groups; and further distances were observed between the Tujia group and Chinese Kazak^9^, Uigur^9^ groups; whereas the larger distances were estimated with Uruguayan^24^, Dane^25^, Central Spanish^26^, Basque^26^, and Hungarian populations^27^. Pairwise populations had small genetic distances, which indicated that they had close genetic relationships or shared a recent common ancestor.

### InDel diversities among populations

Population differentiations for 30 InDels were compared between the Tujia group and other populations previously published based on AMOVA method (*p* < 0.05). As shown in Table 3, the AMOVA comparison results showed significant differences between the Tujia group and Shanghai Han, Beijing Han, Guangdong Han, She, Xibe, South Korean, Tibetan, Yi, Uigur, Kazak, Uruguayan, Hungarian, Basque, Dane, Central Spanish populations at 1, 3, 3, 4, 5, 7, 8, 9, 14, 14, 20, 20, 20, 21 and 22 loci, respectively. The present results demonstrated that the HLD125, HLD99, HLD67, HLD118 loci had relatively high level of genetic variation, with the significant differentiation between Tujia group and other 9, 10, 10 and 11 populations, respectively; while the least differentiation was obtained at the HLD92, HLD101, HLD124 loci with only one pair-wise population. Therefore, allele frequency data obtained at 30 InDels are very important and necessary for forensic application research of different populations.

### Principal component analyses

**Figure 1:**
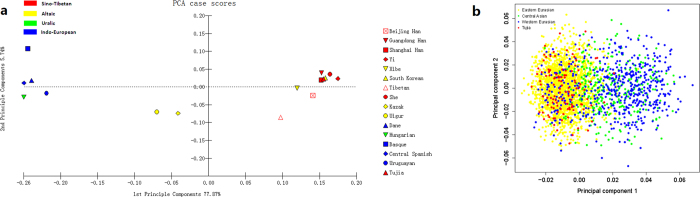
A PCA plot showing the genetic relationships. (**a**) Tujia group and other 15 reference populations. (**b**) Tujia, central Asian, western Eurasian and other eastern Eurasian populations were analyzed at individual level.

On the basis of the allelic frequencies at the same 30 InDels, PCA figures were constructed by MATLAB 2007a (MathWorks Inc., USA) and R statistical software v3.0.2^21^ among the Tujia group and other 15 reference populations. As shown in Fig. 1a, the variance ratio contribution of the first principal component (PC) was about 77.87% of the total variation and the second accounted for 5.74%. In the PCA diagram, the 16 populations were divided into three relatively independent areas inconsistency with their languages family. Ethnic groups with similar language family basically spread closer. The results indicated that there were close relationships between the Tujia group and Chinese Han populations from different regions, as well as She and South Korean groups. Ya *et al*. studied the haplotypes of 17 Y-STR loci and preformed the multidimensional scaling plot which also showed the close relationship between Tujia and Han population^28^; and the similar result was observed in the PCA plot based on the allelic frequencies of HLA-DRB1 locus^29^. The relatively far genetic relationships between Tujia group and Kazak or Uigur group were observed in the PCA map constructed by mtDNA haplogroup frequencies^30^ and in the abovementioned HLA-DRB1 PCA plot^29^, respectively.

The genetic relationships among Tujia, central Asian (Uigur and Kazak populations), western Eurasians (Hungarian, Dane, Basque and Central Spanish populations) and other eastern Eurasians (Shanghai Han, Beijing Han, Guangdong Han, She, Xibe, South Korean, Tibetan, and Yi populations) were also discerned with the aid of abovementioned InDel datasets at the individual level. Results of individual PCA were presented by the plots of the first two PCs (shown in Fig. 1b), which together accounted for 38.82% of the total variation in these populations. The first PC revealed an east-west geographic division within Eurasians. In concrete terms, all eastern Eurasians tended to cluster on the left of PCA plots, whereas western Eurasians formed a separate cluster on the right. The Tujia people were expectedly clustered within eastern Asian group.

### Neighbor-joining phylogenetic reconstruction

**Figure 2:**
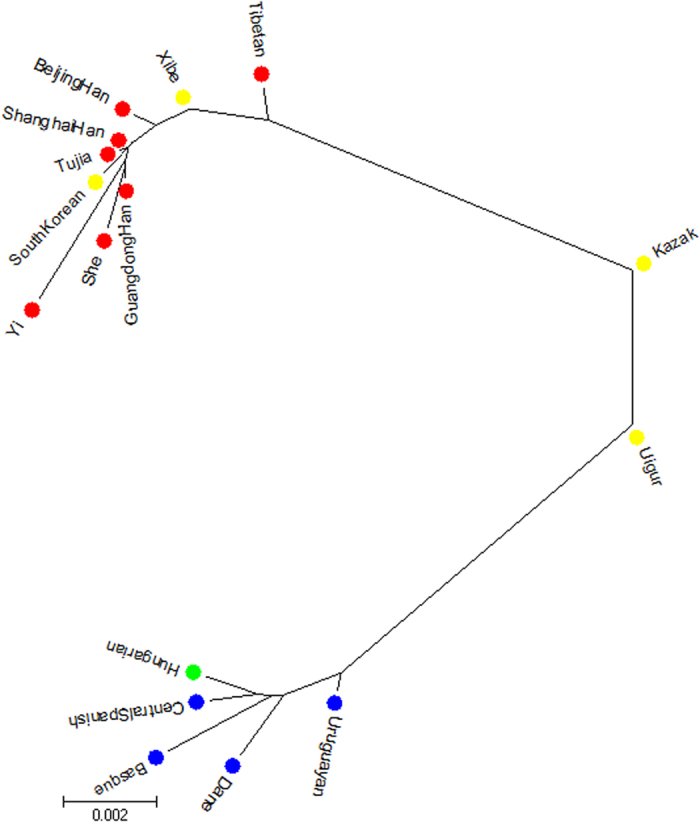
A neighbor-joining phylogenetic tree constructed to analyze phylogenetic relationships from Tujia group and 15 reference populations.

We constructed a neighbor-joining (N-J) phylogenetic tree (shown in Fig. 2). The branch in the upper-left corner contained the nine East Asian populations including Tujia group; whereas in the other branch, Dane, Basque, Central Spanish, Uruguayan, and Hungarian populations were found in the lower-left corner. The Kazak and Uigur groups were in the middle of the above two branches. In previous study, the close relationship between Tujia group and Han population was observed in the N-J dendrogram based on the allelic frequencies of HLA-A locus^31^. The language of Tujia belongs to Tibeto-Burman language system, without written script. Tujias lived with other nationalities like Miao and Han, and many of them can speak Mandarin Chinese and write the Chinese characters. The tight genetic relationship between the Tujia and Han population in Hubei provience was observed based on fifteen STRs, and the present and previous studies indicated that broad genetic exchanges had occurred among them in history^32^.

### Population STRUCTURE analyses

**Figure 3:**
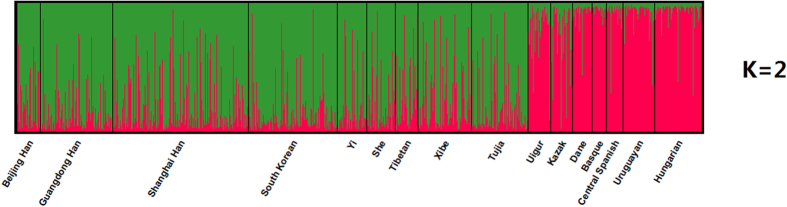
Population STRUCTURE analysis of 16 populations at *K* = 2, which revealed three major clusters.

The STRUCTURE program was used to evaluate the genetic structure of Tujia and other 15 populations. As shown in Fig. 3, at *K* = 2, three clusters were highly visible and easily distinguishable basically by red, green and mixture of the two. When *K* = 2–7 (in Supplementary Fig. 2), the STRUCTURE analyses revealed three major clusters: the first subpopulation of Dane, Basque, Central Spanish, Uruguayan, and Hungarian populations, the second of Kazak and Uigur; the last one of nine East Asian populations including Tujia group. The results presented here were similar to that of the PCA plot and N-J tree. With the increase of *K* values, no further population structures were obtained. We should, just as a precaution, study more ancestry informative InDels in the future in order to subdivide the genetic structure of different ethnic groups in China, and to infer the population origin and ancestral components of an unknown individual.

## Conclusion

In summary, the population data here indicated the 30 InDels had high diversities within the studied group and genetic differentiations among different populations; and could be a useful supplement to the routine detection of autosomal STRs in forensic cases. The PCA plot, N-J tree and STRUCTURE analyses suggested the close relationships between Tujia and Han population in different regions. More ancestry informative InDels and SNPs should be selected and validated to clarify the Tujia ancestral origin.

## Additional Information

**How to cite this article**: Shen, C. *et al*. A 30-InDel Assay for Genetic Variation and Population Structure Analysis of Chinese Tujia Group. *Sci. Rep.* **6**, 36842; doi: 10.1038/srep36842 (2016).

**Publisher’s note**: Springer Nature remains neutral with regard to jurisdictional claims in published maps and institutional affiliations.

## Supplementary Material

Supplementary Information

Supplementary Table 1

## Figures and Tables

**Table 1:** The allelic frequencies and forensic efficiency parameters for 30 InDels in Chinese Tujia ethnic Group (n = 236).

HLD	DIP(−)	DIP(+)	PD	MP	PIC	PE	TPI	HO	HE	*p*
HLD6 (rs1610905)	0.5318	0.4682	0.6164	0.3836	0.3740	0.1989	1.0261	0.5127	0.4980	0.6744
HLD39 (rs17878444)	0.8835	0.1165	0.3519	0.6481	0.1847	0.0343	0.6378	0.2161	0.2059	0.7107
HLD40 (rs2307956)	0.3263	0.6737	0.6028	0.3972	0.3430	0.1030	0.8082	0.3814	0.4396	0.0669
HLD45 (rs2307959)	0.3475	0.6525	0.5937	0.4063	0.3506	0.1595	0.9365	0.4661	0.4535	0.7186
HLD48 (rs28369942)	0.5572	0.4428	0.6072	0.3928	0.3717	0.2067	1.0442	0.5212	0.4935	0.4123
HLD56 (rs2308292)	0.4492	0.5508	0.6061	0.3939	0.3724	0.2107	1.0536	0.5254	0.4948	0.3640
HLD58 (rs1610937)	0.5975	0.4025	0.6101	0.3899	0.3653	0.1802	0.9833	0.4915	0.4810	0.7702
HLD64 (rs1610935)	0.1398	0.8602	0.3997	0.6003	0.2116	0.0493	0.6782	0.2627	0.2406	0.4369
HLD67 (rs1305056)	0.2458	0.7542	0.5373	0.4627	0.3020	0.0809	0.7564	0.3390	0.3707	0.3010
HLD70 (rs2307652)	0.4364	0.5636	0.6080	0.3920	0.3709	0.2028	1.0351	0.5169	0.4919	0.4611
HLD77 (rs1611048)	0.5360	0.4640	0.6323	0.3677	0.3737	0.1696	0.9593	0.4788	0.4974	0.5461
HLD81 (rs17879936)	0.1695	0.8305	0.4451	0.5549	0.2419	0.0588	0.7024	0.2881	0.2815	0.8375
HLD83 (rs2308072)	0.6186	0.3814	0.6086	0.3914	0.3605	0.1662	0.9516	0.4746	0.4718	0.9576
HLD84 (rs3081400)	0.2394	0.7606	0.5307	0.4693	0.2979	0.0829	0.7613	0.3432	0.3642	0.4879
HLD88 (rs8190570)	0.4470	0.5530	0.6476	0.3524	0.3722	0.1319	0.8741	0.4280	0.4944	0.0382
HLD92 (rs17174476)	0.5445	0.4555	0.5998	0.4002	0.3730	0.2231	1.0826	0.5381	0.4960	0.2073
HLD93 (rs2307570)	0.4407	0.5593	0.6332	0.3668	0.3715	0.1595	0.9365	0.4661	0.4930	0.3912
HLD97 (rs17238892)	0.6610	0.3390	0.5947	0.4053	0.3477	0.1467	0.9077	0.4492	0.4481	0.9987
HLD99 (rs2308163)	0.1356	0.8644	0.3723	0.6277	0.2069	0.0284	0.6211	0.1949	0.2344	0.1472
HLD101 (rs2307433)	0.5339	0.4661	0.6442	0.3558	0.3738	0.1467	0.9077	0.4492	0.4977	0.1275
HLD111 (rs1305047)	0.9089	0.0911	0.2809	0.7191	0.1519	0.0173	0.5871	0.1483	0.1656	0.4661
HLD114 (rs2307581)	0.7648	0.2352	0.5262	0.4738	0.2950	0.0829	0.7613	0.3432	0.3597	0.5805
HLD118 (rs16438)	0.0445	0.9555	0.1621	0.8379	0.0814	0.0067	0.5488	0.0890	0.0850	0.8353
HLD122 (rs8178524)	0.7669	0.2331	0.5240	0.4760	0.2936	0.0730	0.7375	0.3220	0.3575	0.2461
HLD124 (rs6481)	0.4174	0.5826	0.6213	0.3787	0.3681	0.1696	0.9593	0.4788	0.4863	0.7924
HLD125 (rs16388)	0.6483	0.3517	0.5928	0.4072	0.3520	0.1662	0.9516	0.4746	0.4560	0.5874
HLD128 (rs2307924)	0.6504	0.3496	0.5933	0.4067	0.3513	0.1628	0.9440	0.4703	0.4547	0.6518
HLD131 (rs1611001)	0.6653	0.3347	0.5856	0.4144	0.3462	0.1595	0.9365	0.4661	0.4454	0.5411
HLD133 (rs2067235)	0.6377	0.3623	0.5970	0.4030	0.3553	0.1696	0.9593	0.4788	0.4621	0.6273
HLD136 (rs16363)	0.4725	0.5275	0.6370	0.3630	0.3742	0.1628	0.9440	0.4703	0.4985	0.3696

DIP(−), frequency of deletion allele; DIP(+), frequency of insertion allele; PD, power of discrimination; MP, matching probability; PIC, polymorphic information content; PE, power of exclusion; TPI, typical paternity index; HO, observed heterozygosity; HE, expected heterozygosity; *p*, probability values for Hardy-Weinberg equilibrium tests.

**Table 2:** *D*_*A*_ distances between Tujia group and other populations based on allelic frequencies of the same set of 30 InDel loci.

Populations	Shanghai Han	Beijing Han	Guangdong Han	Tujia	Tibetan	She	South Korean	Uigur	Kazak	Dane	Basque	Central Spanish	Uruguayan	Hungarian	Yi	Xibe	References
Shanghai Han	*																Wang *et al*.^17^
Beijing Han	0.0011	*															Wei *et al*.^9^
Guangdong Han	0.0006	0.0019	*														Hong *et al*.^18^
Tujia	0.0004	0.0013	0.0007	*													Present study
Tibetan	0.0038	0.0029	0.0055	0.0038	*												Wei *et al*.^9^
She	0.0019	0.0023	0.0015	0.0019	0.0065	*											Wang *et al*.^17^
South Korean	0.0008	0.0024	0.0017	0.0009	0.0038	0.0028	*										Seong *et al*.^23^
Uigur	0.0114	0.0100	0.0118	0.0125	0.0093	0.0133	0.0135	*									Wei *et al*.^9^
Kazak	0.0096	0.0083	0.0100	0.0104	0.0074	0.0112	0.0115	0.0013	*								Wei *et al*.^9^
Dane	0.0264	0.0251	0.0265	0.0272	0.0226	0.0275	0.0288	0.0083	0.0093	*							Friis *et al*.^25^
Basque	0.0270	0.0270	0.0268	0.0281	0.0258	0.0288	0.0287	0.0096	0.0111	0.0048	*						Martin *et al*.^26^
Central Spanish	0.0268	0.0262	0.0269	0.0277	0.0231	0.0285	0.0288	0.0069	0.0085	0.0030	0.0033	*					Martin *et al*.^26^
Uruguayan	0.0240	0.0230	0.0244	0.0250	0.0199	0.0255	0.0258	0.0057	0.0067	0.0039	0.0043	0.0023	*				Saiz *et al*.^24^
Hungarian	0.0271	0.0255	0.0275	0.0281	0.0222	0.0289	0.0295	0.0068	0.0084	0.0026	0.0045	0.0022	0.0021	*			Kis *et al*.^27^
Yi	0.0040	0.0054	0.0038	0.0040	0.0066	0.0051	0.0042	0.0163	0.0133	0.0315	0.0328	0.0323	0.0286	0.0038	*		Zhang *et al*.^16^
Xibe	0.0015	0.0022	0.0023	0.0015	0.0037	0.0032	0.0016	0.0092	0.0068	0.0227	0.0236	0.0226	0.0203	0.0023	0.0052	*	Meng *et al*.^10^

**Table 3:** Pairwise *Fst* and *p* values between the Tujia group and other 15 populations based on AMOVA method.

Loci	Shanghai Han		Beijing Han		Guangdong Han		Tibetan		She		Yi		South Korean		Uigur		Kazak		Xibe		Dane		Basque		Central Spanish		Uruguayan		Hungarian	
	*F*_*ST*_	*p*	*F*_*ST*_	*p*	*F*_*ST*_	*p*	*F*_*ST*_	*p*	*F*_*ST*_	*p*	*F*_*ST*_	*p*	*F*_*ST*_	*p*	*F*_*ST*_	*p*	*F*_*ST*_	*p*	*F*_*ST*_	*p*	*F*_*ST*_	*p*	*F*_*ST*_	*p*	*F*_*ST*_	*p*	*F*_*ST*_	*p*	*F*_*ST*_	*p*
D77	−0.0009	1.0000	0.0067	0.1163	0.0001	0.4467	−0.0023	1.0000	−0.0025	1.0000	0.0388	0.0000	−0.0018	1.0000	−0.0036	1.0000	−0.0039	1.0000	0.0031	0.1525	0.0354	0.0020	0.0246	0.0225	0.0031	0.3030	0.0094	0.0547	−0.0025	1.0000
D45	0.0017	0.2160	−0.0036	1.0000	−0.0011	1.0000	−0.0012	0.7107	−0.0029	1.0000	−0.0032	1.0000	0.0070	0.0372	−0.0034	1.0000	0.0065	0.1496	−0.0013	1.0000	0.0402	0.0000	0.0432	0.0039	0.0244	0.0156	0.0298	0.0020	0.0523	0.0000
D131	−0.0011	1.0000	0.0004	0.4555	−0.0019	1.0000	0.0526	0.0000	−0.0029	1.0000	0.0021	0.2708	−0.0001	0.4868	0.0494	0.0000	0.0248	0.0108	−0.0012	1.0000	0.0695	0.0000	0.0000	0.5044	0.0521	0.0010	0.0708	0.0000	0.1122	0.0000
D70	−0.0015	1.0000	0.0023	0.2972	−0.0018	1.0000	0.0014	0.3441	0.0062	0.1095	0.0012	0.3646	0.0084	0.0127	−0.0015	0.7928	−0.0027	1.0000	0.0036	0.1623	0.0042	0.1926	0.0103	0.1095	0.0005	0.4477	0.0163	0.0205	−0.0010	0.7703
D6	−0.0015	1.0000	−0.0021	1.0000	−0.0011	1.0000	−0.0032	1.0000	0.0078	0.0723	0.0137	0.0274	0.0009	0.2864	0.0091	0.0616	0.0219	0.0147	0.0089	0.0342	0.0025	0.3021	0.0057	0.1760	−0.0013	0.6960	0.0236	0.0000	0.0024	0.2473
D111	−0.0012	1.0000	−0.0038	1.0000	−0.0008	0.8475	−0.0038	1.0000	0.0034	0.2033	−0.0022	1.0000	0.0007	0.3578	0.1552	0.0000	0.1423	0.0000	0.0032	0.1496	0.4417	0.0000	0.4824	0.0000	0.4051	0.0000	0.3809	0.0000	0.4412	0.0000
D58	−0.0015	1.0000	−0.0029	1.0000	0.0065	0.0489	0.0031	0.2405	−0.0032	1.0000	−0.0017	1.0000	0.0080	0.0284	−0.0026	1.0000	−0.0036	1.0000	0.0204	0.0020	0.0464	0.0020	0.0957	0.0000	0.0345	0.0029	0.0027	0.2502	0.0330	0.0000
D56	0.0005	0.3617	−0.0015	0.8113	0.0048	0.0850	0.0085	0.0919	−0.0029	1.0000	0.0298	0.0000	−0.0007	0.8025	0.0016	0.2854	−0.0012	0.6432	0.0025	0.2199	0.0161	0.0352	0.0039	0.2385	0.0771	0.0000	−0.0024	1.0000	0.0348	0.0000
D118	0.0017	0.1994	0.0018	0.3421	0.0085	0.0293	−0.0027	1.0000	0.0581	0.0000	0.0315	0.0000	−0.0014	1.0000	0.4123	0.0000	0.3246	0.0000	0.0117	0.0078	0.5670	0.0000	0.6264	0.0000	0.6200	0.0000	0.5830	0.0000	0.4585	0.0000
D92	−0.0011	1.0000	−0.0033	1.0000	0.0022	0.2033	−0.0017	0.9267	−0.0029	1.0000	−0.0001	0.4790	0.0010	0.2727	0.0052	0.1613	0.0160	0.0156	−0.0006	0.6647	−0.0035	1.0000	−0.0020	0.7439	−0.0024	1.0000	0.0009	0.3314	0.0010	0.3109
D93	−0.0012	1.0000	−0.0032	1.0000	−0.0021	1.0000	0.0058	0.1535	0.0097	0.0499	0.0334	0.0000	0.0030	0.1408	0.0090	0.0821	−0.0015	0.7322	0.0057	0.0919	0.0002	0.4477	0.0060	0.2121	0.0009	0.3803	−0.0023	1.0000	0.0005	0.3744
D99	−0.0015	1.0000	0.0265	0.0078	−0.0002	0.4897	0.0418	0.0049	−0.0015	0.8328	−0.0011	0.6823	0.0112	0.0088	0.1657	0.0000	0.1740	0.0000	−0.0007	0.6843	0.2225	0.0000	0.1550	0.0000	0.1914	0.0000	0.1529	0.0000	0.1998	0.0000
D88	0.0014	0.1975	0.0036	0.2444	0.0024	0.1672	0.0075	0.1036	−0.0027	1.0000	−0.0026	1.0000	0.0009	0.3578	0.0071	0.1124	−0.0016	0.7302	−0.0011	0.8416	0.0095	0.1007	0.0399	0.0059	0.0285	0.0156	0.0006	0.4096	−0.0015	1.0000
D101	−0.0015	1.0000	−0.0028	1.0000	−0.0018	1.0000	−0.0033	1.0000	0.0107	0.0635	−0.0016	0.8856	0.0004	0.4027	0.0292	0.0049	0.0081	0.1075	−0.0013	1.0000	0.0144	0.0557	−0.0032	1.0000	−0.0002	0.5220	0.0016	0.3118	−0.0019	1.0000
D67	0.0104	0.0108	0.0037	0.1896	0.0008	0.3265	0.0591	0.0010	−0.0006	0.5494	0.0030	0.2542	0.0106	0.0166	0.0316	0.0010	0.0729	0.0000	0.0017	0.2708	0.0564	0.0010	0.1682	0.0000	0.0351	0.0059	0.0711	0.0000	0.0630	0.0000
D83	−0.0006	0.7331	0.0244	0.0098	−0.0010	1.0000	0.0008	0.4330	0.0186	0.0127	0.0294	0.0020	−0.0011	1.0000	−0.0018	0.9247	−0.0021	1.0000	−0.0009	0.8035	0.0563	0.0010	0.0570	0.0000	0.0348	0.0068	0.0059	0.0987	0.0099	0.0303
D114	0.0020	0.1975	0.0040	0.1818	−0.0017	1.0000	0.0008	0.4458	−0.0021	1.0000	−0.0031	1.0000	0.0047	0.0821	0.0528	0.0000	0.0828	0.0000	0.0159	0.0068	0.0402	0.0010	0.0156	0.0792	0.1344	0.0000	0.0664	0.0000	0.0273	0.0000
D48	0.0053	0.0577	−0.0008	0.5973	0.0043	0.0792	−0.0035	1.0000	0.0053	0.1427	0.0082	0.0743	0.0070	0.0362	−0.0005	0.5279	−0.0035	1.0000	0.0004	0.3617	0.1026	0.0000	0.0112	0.1017	0.0213	0.0196	0.0124	0.0352	−0.0002	0.5435
D124	0.0019	0.1877	−0.0027	1.0000	0.0082	0.0284	0.0067	0.1329	0.0067	0.1075	−0.0028	1.0000	−0.0003	0.5552	0.0053	0.1711	−0.0038	1.0000	−0.0021	1.0000	−0.0026	1.0000	0.0157	0.0587	0.0043	0.2072	0.0043	0.1789	0.0042	0.1241
D122	−0.0015	1.0000	−0.0030	1.0000	0.0017	0.2287	0.0544	0.0000	0.0213	0.0098	−0.0031	1.0000	0.0008	0.3099	0.0406	0.0020	0.0379	0.0010	0.0020	0.2307	0.0496	0.0000	0.0204	0.0430	0.0639	0.0000	0.0855	0.0000	0.1353	0.0000
D125	−0.0014	1.0000	0.0161	0.0235	0.0000	0.5064	0.0155	0.0313	0.0019	0.3109	0.0089	0.0547	−0.0016	1.0000	0.0511	0.0000	0.0241	0.0059	0.0046	0.1105	0.0698	0.0000	0.0270	0.0137	0.0223	0.0156	0.0206	0.0049	0.0648	0.0000
D64	0.0010	0.2669	−0.0026	1.0000	−0.0003	0.5640	0.0032	0.2219	−0.0027	1.0000	0.0034	0.1730	−0.0015	1.0000	0.0900	0.0000	0.0849	0.0000	0.0276	0.0000	0.2012	0.0000	0.2841	0.0000	0.1842	0.0000	0.0928	0.0000	0.2286	0.0000
D81	−0.0013	1.0000	−0.0023	1.0000	0.0022	0.2111	−0.0032	1.0000	0.0015	0.3597	0.0086	0.0596	0.0060	0.0332	0.0385	0.0000	0.0484	0.0000	−0.0017	1.0000	0.2773	0.0000	0.2284	0.0000	0.3333	0.0000	0.2985	0.0000	0.2766	0.0000
D136	−0.0015	1.0000	−0.0006	0.5503	0.0043	0.0899	−0.0025	1.0000	0.0042	0.1936	0.0566	0.0000	0.0042	0.0811	−0.0036	1.0000	−0.0009	0.6012	−0.0018	1.0000	−0.0021	0.9228	0.0529	0.0000	−0.0031	1.0000	−0.0007	0.6139	0.0017	0.2825
D133	−0.0015	1.0000	−0.0033	1.0000	−0.0015	1.0000	0.0132	0.0362	0.0028	0.2581	0.0001	0.4379	−0.0013	1.0000	0.0034	0.2502	0.0125	0.0567	0.0018	0.2415	0.1098	0.0000	0.1181	0.0000	0.1272	0.0000	0.0610	0.0000	0.0844	0.0000
D97	−0.0016	1.0000	−0.0004	0.5200	−0.0007	0.7459	−0.0030	1.0000	−0.0013	0.7527	−0.0019	1.0000	−0.0016	1.0000	0.0041	0.2160	−0.0024	1.0000	0.0004	0.3617	0.0703	0.0000	0.1333	0.0000	0.0631	0.0000	0.0596	0.0000	0.0482	0.0000
D128	0.0010	0.3089	−0.0022	1.0000	−0.0017	1.0000	0.0014	0.3372	0.0000	0.4809	0.0931	0.0000	−0.0018	1.0000	0.0104	0.0596	0.0033	0.2796	−0.0020	1.0000	0.0101	0.1007	0.0469	0.0029	0.0175	0.0244	0.0166	0.0078	0.0348	0.0000
D39	−0.0011	1.0000	−0.0012	0.6872	0.0006	0.3724	0.0667	0.0000	−0.0011	0.7126	−0.0005	0.6158	−0.0011	1.0000	0.1172	0.0000	0.0423	0.0020	0.0034	0.1349	0.1109	0.0000	0.0886	0.0000	0.2282	0.0000	0.2037	0.0000	0.1837	0.0000
D40	0.0027	0.1251	0.0002	0.4575	0.0003	0.4409	0.0353	0.0010	0.0003	0.4282	0.0198	0.0098	0.0032	0.1496	0.0325	0.0029	0.0377	0.0020	0.0033	0.1652	0.1218	0.0000	0.1089	0.0000	0.1310	0.0000	0.0774	0.0000	0.1087	0.0000
D84	−0.0010	1.0000	0.0014	0.3724	0.0021	0.1926	0.0103	0.0733	0.0066	0.1202	−0.0007	0.5816	−0.0017	1.0000	0.0529	0.0000	0.0046	0.1525	0.0028	0.1711	0.1243	0.0000	0.0699	0.0000	0.0534	0.0010	0.0982	0.0000	0.0916	0.0000
