# Supplementary material for: A 30-InDel Assay for Genetic Variation and Population Structure Analysis of Chinese Tujia Group
Source: Sci Rep. 2016 Nov 11;6:36842. doi: 10.1038/srep36842 (PMC5104975; doi:10.1038/srep36842)

**Title:**

**A 30-InDel Assay for Genetic Variation and Population Structure Analysis of Chinese Tujia Group**

**Author:**

**Chunmei Shen**^a,b*^**, Bofeng Zhu**^c,d*^**, Tianhua Yao**^d,e^**, Zhidan Li**^e,f^**, Yudang Zhang**^g^**, Jiangwei Yan**^h^**, Bo Wang**^i^**, Xiaohua Bie**j,
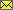
, **, Fadao Tai**^b^

^a^Blood Center of Shaanxi Province, Xi'an, Shaanxi 710061, P.R. China.

^b^Institute of Brain and Behavioral Sciences, College of Life Sciences, Shaanxi Normal University, Xi'an, Shaanxi 710062, P.R. China.

^c^Department of Forensic Genetics, School of Forensic Medicine, Southern Medical University, Guangzhou 510515, P.R. China.

^d^Key Laboratory of Shaanxi Province for Craniofacial Precision Medicine Research, College of Stomatology, Xi'an Jiaotong University, Xi'an, Shaanxi 710004, P. R. China.

^e^Clinical Research Center of Shaanxi Province for Dental and Maxillofacial Diseases, College of Stomatology, Xi’an Jiaotong University, Xi'an, Shaanxi 710004, P. R. China.

^f^Department of Endodontics, Stomatological Hospital, Xi'an Jiaotong University, Xi'an, Shaanxi 710004, P. R. China.

^g^Institute of Forensic Science of Anhui Public Security Department, Hefei, Anhui [230061](http://www.youbian.com/230061/), P. R. China.

^h^Key Laboratory of Genome Sciences, Beijing Institute of Genomics, Chinese Academy of Sciences, Beijing 100101, P. R. China.

^i^College of Biological Technology, Xi'an University, Xi'an, Shaanxi 710065, P. R. China.

^j^Department of Neurosurgery, Hong-hui Hospital, Xi’an Jiaotong University College of Medicine,  Xi’an, Shaanxi 710054, P. R. China

^*^ These authors contributed equally to this work.

**Correspondence**
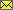
**:**

Dr. Xiao-Hua Bie, Department of Neurosurgery, Hong-hui Hospital, Xi’an Jiaotong University College of Medicine,  Xi’an, Shaanxi 710054, P. R. China

E-mail: [biexiaohua1@126.com](mailto:biexiaohua1@126.com)

Fig.S1.The LD results of pairwise 30 InDels using the SNPAnalyzer version 2.0


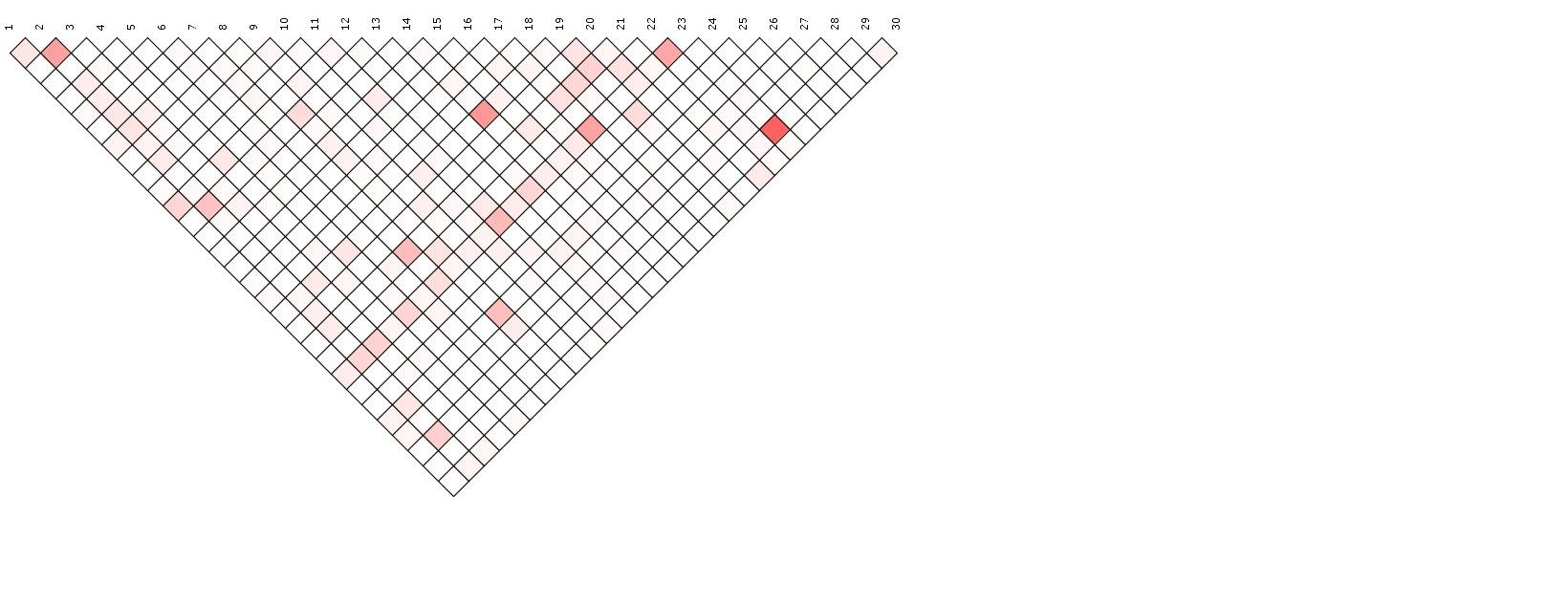


Fig.S2. Population STRUCTURE analysis of 16 populations at *K*=2-7


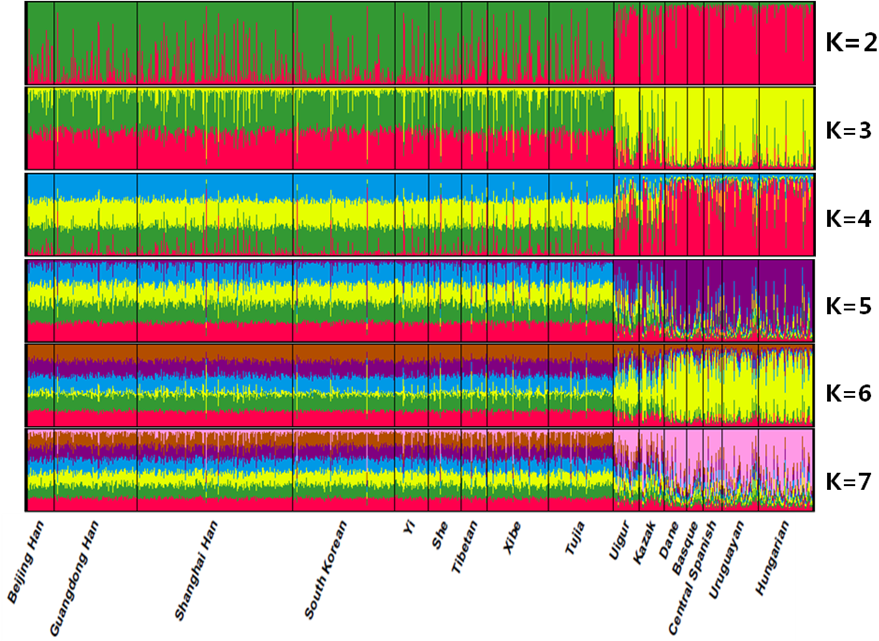

Supplement: Supplementary Information [file srep36842-s1.doc]
